# Quantitative Oculomotor Assessment in Hereditary Ataxia: Systematic Review and Consensus by the Ataxia Global Initiative Working Group on Digital-motor Biomarkers

**DOI:** 10.1007/s12311-023-01559-9

**Published:** 2023-04-28

**Authors:** Pilar Garces, Chrystalina A. Antoniades, Anna Sobanska, Norbert Kovacs, Sarah H. Ying, Anoopum S. Gupta, Susan Perlman, David J. Szmulewicz, Chiara Pane, Andrea H. Németh, Laura B. Jardim, Giulia Coarelli, Michaela Dankova, Andreas Traschütz, Alexander A. Tarnutzer

**Affiliations:** 1 Roche Pharma Research and Early Development, Neuroscience and Rare Diseases, Roche Innovation Center Basel, Basel, Switzerland; 2 NeuroMetrology Lab, Nuffield Department of Clinical Neurosciences, Clinical Neurology, Medical Sciences Division, University of Oxford, Oxford, OX3 9DU UK; 3 Institute of Psychiatry and Neurology, Warsaw, Poland; 4 Department of Neurology, University of Pécs, Medical School, Pécs, Hungary; 5 Department of Otology and Laryngology and Department of Neurology, Harvard Medical School, Boston, MA USA; 6 Department of Neurology, Massachusetts General Hospital, Harvard Medical School, Boston, MA USA; 7 University of California Los Angeles, Los Angeles, California USA; 8 Balance Disorders and Ataxia Service, Royal Victoria Eye and Ear Hospital, East Melbourne, Melbourne, VIC 3002 Australia; 9 The Florey Institute of Neuroscience and Mental Health, Parkville, Melbourne, VIC 3052 Australia; 10 Department of Neurosciences and Reproductive and Odontostomatological Sciences, University of Naples “Federico II”, Naples, Italy; 11 Nuffield Department of Clinical Neurosciences, University of Oxford, Oxford, UK; 12 Oxford Centre for Genomic Medicine, Oxford University Hospitals NHS Trust, Oxford, UK; 13 Departamento de Medicina Interna, Universidade Federal do Rio Grande do Sul, Porto Alegre, Brazil; 14 Serviço de Genética Médica/Centro de Pesquisa Clínica e Experimental, Hospital de Clínicas de Porto Alegre, Porto Alegre, Brazil; 15 Sorbonne Université, Institut du Cerveau - Paris Brain Institute - ICM, Inserm U1127, CNRS UMR7225, Paris, France; 16 Department of Genetics, Neurogene National Reference Centre for Rare Diseases, Pitié-Salpêtrière University Hospital, Assistance Publique, Hôpitaux de Paris, Paris, France; 17 Department of Neurology, Centre of Hereditary Ataxias, 2nd Faculty of Medicine, Charles University and Motol University Hospital, Prague, Czech Republic; 18 Research Division “Translational Genomics of Neurodegenerative Diseases”, Hertie-Institute for Clinical Brain Research and Center of Neurology, University of Tübingen, Tübingen, Germany; 19 German Center for Neurodegenerative Diseases (DZNE), University of Tübingen, Tübingen, Germany; 20 Neurology, Cantonal Hospital of Baden, 5404 Baden, Switzerland; 21 Faculty of Medicine, University of Zurich, Zurich, Switzerland

**Keywords:** Oculomotor, Vestibular, Eye movement recordings, Hereditary ataxia, Systematic review, Recommendations

## Abstract

Oculomotor deficits are common in hereditary ataxia, but disproportionally neglected in clinical ataxia scales and as outcome measures for interventional trials. Quantitative assessment of oculomotor function has become increasingly available and thus applicable in multicenter trials and offers the opportunity to capture severity and progression of oculomotor impairment in a sensitive and reliable manner. In this consensus paper of the Ataxia Global Initiative Working Group On Digital Oculomotor Biomarkers, based on a systematic literature review, we propose harmonized methodology and measurement parameters for the quantitative assessment of oculomotor function in natural-history studies and clinical trials in hereditary ataxia. MEDLINE was searched for articles reporting on oculomotor/vestibular properties in ataxia patients and a study-tailored quality-assessment was performed. One-hundred-and-seventeen articles reporting on subjects with genetically confirmed (*n*=1134) or suspected hereditary ataxia (*n*=198), and degenerative ataxias with sporadic presentation (*n*=480) were included and subject to data extraction. Based on robust discrimination from controls, correlation with disease-severity, sensitivity to change, and feasibility in international multicenter settings as prerequisite for clinical trials, we prioritize a core-set of five eye-movement types: (i) pursuit eye movements, (ii) saccadic eye movements, (iii) fixation, (iv) eccentric gaze holding, and (v) rotational vestibulo-ocular reflex. We provide detailed guidelines for their acquisition, and recommendations on the quantitative parameters to extract. Limitations include low study quality, heterogeneity in patient populations, and lack of longitudinal studies. Standardization of quantitative oculomotor assessments will facilitate their implementation, interpretation, and validation in clinical trials, and ultimately advance our understanding of the evolution of oculomotor network dysfunction in hereditary ataxias.

**Supplementary Information:**

The online version contains supplementary material available at 10.1007/s12311-023-01559-9.

## Introduction

Patients with hereditary ataxia present with a broad range of symptoms and clinical findings, including deficits in stance and gait, limb coordination, speech, swallowing, and also mood and cognition [1, 2]. Oculomotor deficits are also frequently observed in this population and have the advantage over other motor manifestations in that they are relatively well studied and not confounded by significant inertia or musculoskeletal factors [3–6]. Whereas different types of eye movements have distinct anatomical substrates and their alterations constitute functional readouts of underlying brain pathophysiology (see Leigh and Zee for an in-depth review [7]), specific ataxias often affect more than one brainstem or cerebellar circuit. While traditionally electronystagmography and scleral search coils were the most frequently used approaches to record eye movements, video-oculography (VOG) and infrared systems have become the preferred method due to high recording quality, tolerability, and easy handling [8]. Both the type and the pattern of oculomotor abnormalities may facilitate the differential diagnosis in such patients and thus may allow for a rapid and targeted diagnostic workup [9, 10]. For example, very slow saccades to targets in the context of a dominant family history are suggestive of spinocerebellar ataxia type 2 (SCA2) [11], and bilateral vestibular loss-of-function in the setting of cerebellar ataxia with sensory neuronopathy may point to replication factor complex subunit 1–related disease [12]. Importantly, oculomotor parameters seem to be sensitive and objective biomarkers to monitor disease progression and treatment effects in clinical trials [13, 14].

The identification of the most valuable oculomotor parameters along with their appropriate acquisition paradigms for use in clinical trials is particularly challenging. These parameters are variable and have been assessed and quantified in many different hereditary ataxias [15], but most studies have been single-center without established best practices or standardized protocols. Ideal oculomotor parameters should be easy and reliably measurable in a standardized multi-center setting, and, most importantly, should be sensitive to detect the presence of early disease, disease progression, and the effects of treatment interventions. Noteworthy, the optimal selection of parameters depends on the focus of the research question (distinguishing between diagnostic accuracy studies, longitudinal observational studies, and treatment-response studies) as well as the disease population studied.

The aim of this work, which is an undertaking of the Ataxia Global Initiative (AGI [16]) working group on digital-motor biomarkers, is to propose a core set of quantitative oculomotor parameters for clinical studies of hereditary ataxias. A particular focus is on identifying those parameters (a) that are the most feasible and (b) applicable in multi-center trial settings, (c) that have shown significant discriminatory power for identifying affected individuals from healthy or disease controls, and (d) whose validity has been demonstrated by correlations with other validated measurements of disease severity. Along with the proposed core set of paradigms and parameters, we will propose guidelines for measurement standardization. To achieve these aims, we performed a systematic review of the published literature on quantitative oculomotor testing in ataxia and assessed the suitability of identified parameters regarding the four criteria above. Based on a stepwise review and consensus process discussed extensively with the oculomotor working group, we provide guidelines for the standardized acquisition of a core set of oculomotor assessments, and provide recommendations on the quantitative parameters to derive.

## Material and Methods

### Data Sources and Searches

We searched MEDLINE (via PubMed) for articles using words and controlled-vocabulary terms related to research studies reporting on oculomotor and/or vestibular properties in ataxia patients. A detailed description of the search strategy is available in Appendix 1. Our search was updated through May 13, 2021. No registration on PROSPERO was made.

### Study Selection

Identified articles were reviewed and selected by two independent raters (PG, AAT) using pre-determined inclusion criteria and a structured protocol (see Appendix 1). We focused on studies reporting on quantitative oculomotor and/or vestibular testing in patients with hereditary ataxia, either confirmed or suspected. However, we included studies reporting on degenerative ataxias with sporadic presentation as well. In order to understand the spectrum of oculomotor/vestibular paradigms used in the past in patients presenting with ataxia, we wanted to be as inclusive as possible and thus not to omit findings from patients presenting with ataxia from a non-hereditary origin. We calculated inter-rater agreement on full-text inclusion using Cohen’s kappa [17].

### Data Extraction and Quality Assessment for Studies Reporting on Oculomotor Findings in Ataxia

A quality assessment of included studies was performed by two independent reviewers (PG, AAT) based on eight predefined quality criteria covering items related to (i) the study-cohort, (ii) data acquisition, and (iii) data analysis in studies reporting on oculomotor findings in ataxia, and included an evaluation for risk-of-bias for assessing test results. An overall study quality rating (high, moderate, or low) was derived from this quality assessment (see Appendix 2 for details). In brief, studies were considered “high quality” if they (i) included patients with genetically confirmed hereditary ataxia, (ii) included age-matched healthy control groups (confirmed on clinical examination, except for longitudinal or treatment-response trials), (iii) implemented a prespecified recording protocol, (iv) provided sufficient detail of recorded parameters to allow reproduction, (v) used appropriate (i.e., with sufficiently high-resolution and recording frequency) eye-movement recording devices, (vi) reported on the data analysis performed with sufficient detail to allow reproduction, (vii) were based on normative values retrieved from an appropriate control group, and (viii) had a low risk of bias for assessing the test results. If no genetic testing was available, a positive family history with a clear pattern of inheritance (autosomal dominant, autosomal recessive, X-linked recessive) or established and specific diagnostic biomarkers were a necessary requirement to define studies as “moderate quality.” We did not exclude studies from the review based on the rated study quality.

Data extraction was jointly performed by two reviewers (PG, AAT). We did not contact study investigators to retrieve additional information. Information extracted from each eligible article included the type of study conducted (e.g., case-control studies or observational studies), the number of research participants, the underlying diagnoses, and the actual oculomotor paradigms performed. We extracted detailed information related to the recording device used, the experimental paradigm(s) performed, and the patient cohort(s) studied. This study is reported in accordance with PRISMA guidelines [18].

### Data Synthesis, Parameter, and Paradigm Selection Process

Based on the systematic literature review, consensus was sought for a recommendation of experimental paradigms and quantitative parameters suitable for validation as outcome measures for clinical trials in ataxia. Prioritization considered how often a given experimental paradigm was investigated in different ataxia cohorts, but was ultimately based on (i) the reported robustness to discriminate oculomotor abnormalities in ataxia from healthy or disease control populations; (ii) if validating data was available reporting on the correlation with disease severity, or sensitivity to detect within-subject changes in observational or interventional studies; and (iii) the feasibility of data collection in the setting of large, international, multicenter clinical trials. The existence of standardized recording procedures that can be completed with commercial, readily available devices and software, ideally using a single device for all paradigms, was a key requirement. Paradigms requiring technically demanding, non-standardized, or non-scalable equipment (including rotating chairs, vestibular-evoked myogenic potentials) were discarded.

An initial proposal was designed by a subgroup panel (“parameter validation core group,” *n*=7 participants) after reviewing all studies that reported on specific oculomotor paradigms. The paradigms that met the selection criteria (i)–(iii) as defined above were prioritized, and proposed detailed recommendations on the acquisition setup (e.g., the type of recording device), the stimulation paradigm, and the extraction of quantitative parameters for each paradigm, selecting ranges of parameters that had been successfully implemented in the reviewed studies. The proposal was then critically reviewed and updated by all members of the oculomotor subgroup of the AGI working group on digital-motor biomarkers, and by an expert neuro-otologist not involved in the working group. This was an iterative process until all members of the working group agreed on the proposal.

## Results

### Systematic Literature Review: Included Studies, Study Quality, and Study Goals

**Fig. 1:**
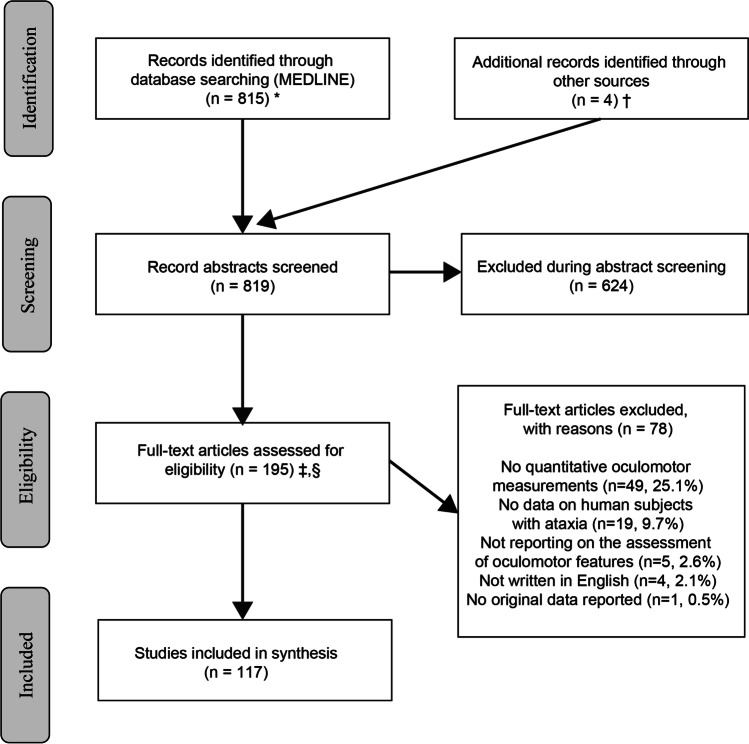
*MEDLINE was accessed via PubMed. ^†^Hand search of citation lists from selected studies and investigator files identified 4 additional manuscripts for review. ^‡^Abstracts coded as “yes” or “maybe” by at least one reviewer were included in full-text review. ^§^After full-text evaluation by two reviewers, any differences were resolved by discussion and—if needed—adjudication by a third, independent reviewer

**Fig. 2:**
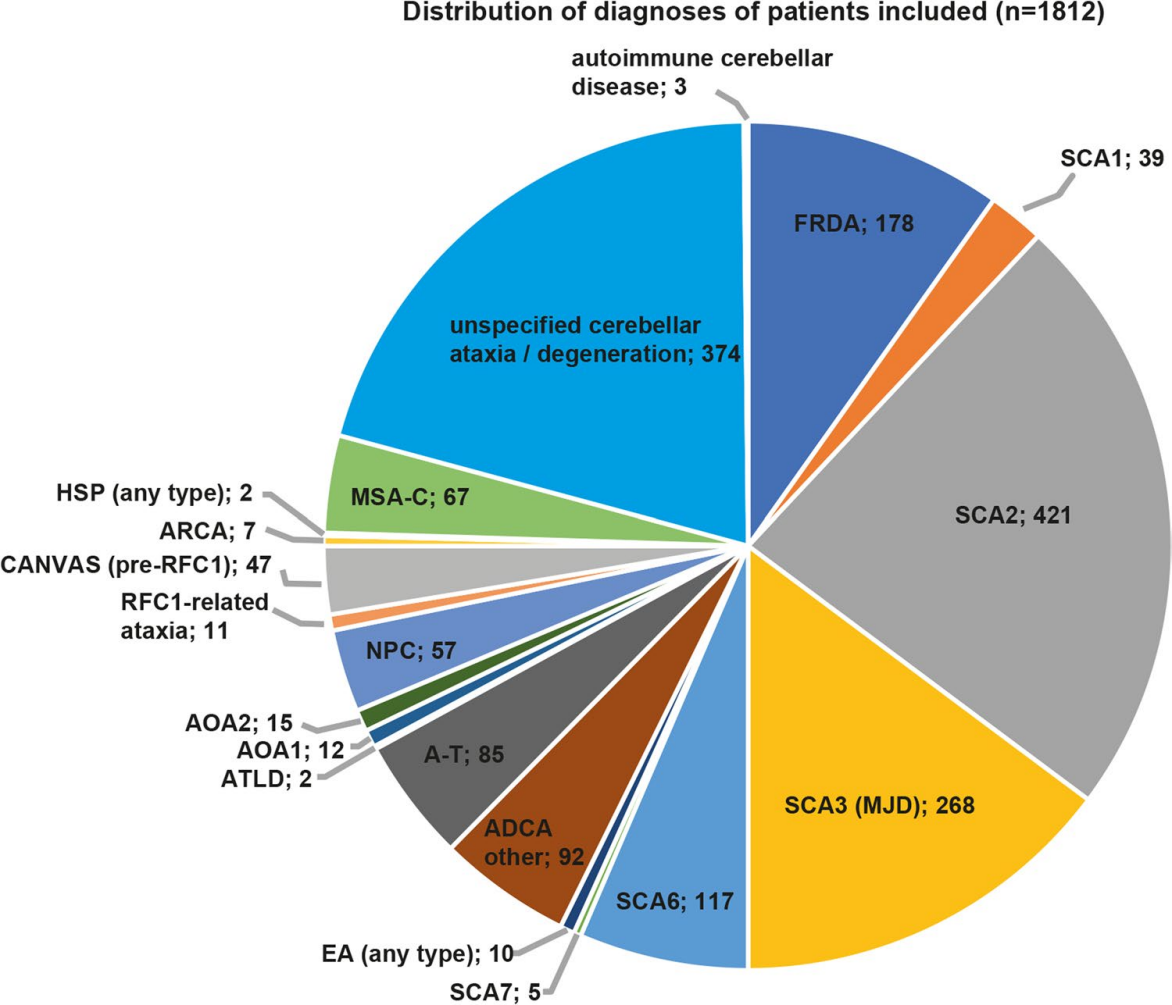
Number of patients with specific ataxia syndromes as reported from all included manuscripts. Abbreviations: ADCA, autosomal-dominant cerebellar ataxia; AOA, ataxia ocular motor apraxia; ARCA, autosomal-recessive cerebellar ataxia; A-T, ataxia telangiectasia; ATLD, ataxia-telangiectasia like disease; CANVAS, cerebellar ataxia, neuropathy, vestibular areflexia syndrome; EA, episodic ataxia; FRDA, Friedreich Ataxia, SCA, spinocerebellar ataxia; HSP, hereditary spastic paraparesis; MJD, Machado-Joseph-Disease (=SCA3); MSA-C, multisystem atrophy type C; RFC, replication factor C subunit 1

Our search identified 819 unique citations, of which 624 (76.2%) were excluded at the abstract level (see Fig. 1—PRISMA flow-chart). Two independent raters had excellent initial agreement on inclusion of full-text manuscripts (kappa value 0.89 (95% CI=0.85–0.94), see Appendix 1). After resolving initial disagreements in the assessment at the full-text level, 117/195 (60.0%) studies were considered eligible, representing 14.3% of the total number of studies. Included studies (*n*=117) reported on 1812 unique patients with either genetically confirmed ataxia (*n*=1134), suspected hereditary ataxia based on family history or biomarkers (*n*=198), and other sporadic or degenerative ataxias (*n*=480). Among genetically confirmed (or suspected) hereditary ataxias, Friedreich Ataxia (FRDA) (*n*=178), spinocerebellar ataxias (SCA) (most often SCA2 (*n*=421), SCA3 (*n*=268), and SCA6 (*n*=117)), and ataxia telangiectasia (A-T) (*n*=85) were most frequently reported (see Fig. 2 for distribution of specific disorders). In twenty-eight studies, a diagnosis of sporadic (degenerative) ataxia was based on the clinical syndrome and patient history (including age at symptom onset and symptom duration), and imaging findings (describing cerebellar/combined cerebellar and brainstem atrophy) (for epidemiologic details, see Table 1 and supplementary Table S1 in Appendix 3).

**Table 1:** Overview of study design and clinical population across studies

	Studies (*n*)	Patients (*n*)
Sex*		
Women	74	638
Men	72	695
Unclear	34	475
Total	117	1812
Study design—time line		
Retrospective	10	73
Prospective	106	1730
Unclear	1	9
Study design—location		
Monocentric	111	1763
Multicentric	6	49
Study type		
Case series	31	312
Case-control study	68	1236
Single case reports	7	7
Observational study	2	112
Randomized controlled treatment study	2	71
Non-randomized treatment study	7	74
Purpose of eye movement recordings		
Characterization of deficits	94	1356
For monitorization of treatment response	11	147
For differential diagnosis (diagnostic accuracy evaluation)	12	309
Patient population studied		
Genetically confirmed hereditary cerebellar ataxia	51	973
Genetically or biochemically confirmed hereditary cerebellar ataxia	19	157
Cohort with both hereditary and non-hereditary cerebellar ataxia	19	411
Early-onset cerebellar ataxia (first symptoms before age 25 years)	2	6
Late-onset cerebellar ataxia	5	103
Other clinical findings suggestive of cerebellar ataxia§	8	88
Cerebellar ataxia (not further defined)	10	71
Acute-onset cerebellar ataxia^#^	1	1
Subacute-onset cerebellar ataxia^†^	2	2

*Missing data in 34 studies (representing 475 patients)

^§^This included downbeat nystagmus [32, 54], cerebellar ataxia neuropathy and vestibular-areflexia [27, 52, 55–57] and progressive ataxia and palatal tremor [58]

^#^Single case with suspected thiamine deficiency [59]

^†^Two single cases (one case with confirmed autoimmune anti-GAD-antibody-associated cerebellar ataxia [37], one case with opsoclonus and cerebellar dysfunction of unknown origin [60])

The overall quality with regard to reporting oculomotor testing in ataxia was rated as “low” in the majority of studies (*n*=69; 59%), whereas a minority of ratings were “moderate” (*n*=26; 22%) or “high” (*n*=22; 19%). The risk of bias was judged “high” in 18 studies, “unclear” in 12 studies, and “low” in the remaining 87 studies (see supplementary Table S2 in Appendix 3). Reasons for low-quality ratings (multiple reasons possible) were most often related to patient selection (lacking genetically confirmed diagnosis, positive family history with clear pattern of inheritance, or established biomarkers; *n*=41), control group selection (being non-age matched and providing no health status; *n*=31), statistical analysis (not providing any information on statistical analyses performed; *n*=32), and unclear risk-of-bias for analysis of the test results (*n*=12).

The sample size in the studies included here ranged from single case reports to larger prospective studies containing up to 82 SCA2 patients and 80 controls [19]. The primary focus of the vast majority of studies was the phenotypic characterization of oculomotor abnormalities in the respective population (*n*=94), being cross-sectional in all but two observational studies that used eye movement recordings to monitor disease progression over period of 12 months [19] and 5 years [20], respectively. Very few studies used oculomotor testing for monitoring treatment responses (*n*=11), or evaluated the value of oculomotor paradigms to differentiate between genetically stratified ataxias (*n*=12) as, e.g., SCA1-3 and FRDA [21].

### Setup, Paradigms, and Quantitative Parameters

The studies reported here included a broad range of oculomotor paradigms (with or without fixation) that were obtained with distinct recording techniques. The most frequently assessed oculomotor paradigms were visually guided saccadic eye movements (SEMs; *n*=73), pursuit eye movements (PEMs; *n*=46), and eccentric gaze holding to assess gaze-evoked nystagmus (GEN; 32) (see Table 2 for details). Among the vestibular assessments, rotational vestibulo-ocular reflex (VOR) testing (*n*=29), VOR-suppression testing (*n*=24), and quantitative head-impulse testing (qHIT; *n*=17) were most often reported (see Table 3 for details).

**Table 2:** Frequency of investigation of oculomotor paradigms across studies

	Stimulus motion plane					
	Studies (*n*)			Subjects (*n*)		
	Horizontal	Vertical	Horizontal and vertical	Horizontal	Vertical	Horizontal and vertical
Pursuit						
Pursuit eye movements	27	0	19	479	0	344
Saccadic eye movements (SEM)						
Visually guided SEM	47	1	25	896	73	304
Memory-guided SEM	4	0	0	99	0	0
Antisaccades	5	0	3	96	0	98
Other SEM	1	0	2	2	0	19
Optokinetic stimulus						
Optokinetic nystagmus	18	0	4	342	0	20
Gaze-holding						
Gaze-evoked nystagmus	14	0	18	152	0	457
Rebound nystagmus	8	0	5	130	0	97
	Eye movement recording plane					
	Studies (*n*)			Subjects (*n*)		
	Horizontal	Vertical	Horizontal and vertical	Horizontal	Vertical	Horizontal and vertical
Fixation instability						
Saccadic intrusions	9	0	18	124	0	396
Gaze-holding						
Spontaneous nystagmus	4	0	25	101	0	454
Triggered nystagmus						
Head-shaking nystagmus	1	0	0	48	0	0
Hyperventilation-induced nystagmus	0	0	1	0	0	11
Positional nystagmus	0	0	5	0	0	103

**Table 3:** Frequency of investigation of vestibular paradigms across studies

	Stimulus motion plane					
	Studies (*n*)			Subjects (*n*)		
	Horizontal	Vertical	Horizontal and vertical	Horizontal	Vertical	Horizontal and vertical
Vestibulo-ocular reflex (VOR)						
VOR rotational	27	0	2	393	0	4
VOR decay Tc	7	0	1	80	0	4
VVOR rotational	11	0	2	107	0	31
VOR translational	2	0	0	17	0	0
OVAR	N/A	N/A	1	N/A	N/A	10
VOR suppression	23	0	1	341	0	4
qHIT	7	0	10	130	0	101
Caloric irrigation	12	N/A	N/A	152	N/A	N/A
Dynamic visual acuity	1	0	0	27	0	0
	No specific stimulus motion plane					
	Studies (*n*)	Subjects (*n*)				
VEMPs						
oVEMPs	4	27				
cVEMPs	7	64				
Behavioral tasks						
Self-motion decay Tc	1	8				
Subjective visual vertical	2	18				

*Abbreviations*: *N/A* not applicable, *OVAR* off-vertical axis rotation, *qHIT* quantitative head-impulse test, *Tc* time constant, *VEMP* vestibular evoked myogenic potentials, *oVEMP* ocular VEMP, *cVEMP* cervical VEMP, *VOR* vestibulo-ocular reflex, *VVOR* vision-enhanced VOR

The most common recording techniques were electro-oculography (EOG, *n*=40 studies and *n*=915 or 50% of patients), and VOG (including infrared oculography, *n*=43 studies and *n*=639 or 35% of patients). Scleral search coil recordings were obtained in a minority of patients (*n*=148; 8%) as illustrated in Table 4. From the 117 studies included, eye movement recordings were restricted to the horizontal plane in 56 studies (reporting on 972 patients), whereas in 58 studies (reporting on 803 patients) both the horizontal and vertical plane were considered. Recordings were binocular in 860 patients, whereas 408 patients had monocular recordings only and no information was provided in the remaining 544 patients. Recording frequency of devices used to quantify eye movement responses ranged between 30 and 1000Hz, strongly depending on the methodology used. Whereas for search coil recordings and EOG recording frequencies were generally 100Hz or above, VOG devices operated at frequencies in the range of 60 to 500Hz.

**Table 4:** Recording setup and normative values across studies

	Studies (*n*)	Subjects (*n*)
Plane of eye movement recordings		
horizontal plane only	56	972
vertical plane only	0	0
both horizontal and vertical plane	58	803
no eye movement data collected	3	37
Number of eyes recorded		
One eye	41	408
Both eyes	41	860
Unclear	35	544
Technique used for eye movement recordings		
Scleral search coils	23	148
Electro-oculography (EOG)	40	915
Video-oculography (incl. infrared) (VOG)	43	639
Mixed (VOG and/or search coils)	3	33
Mixed (EOG or search coils)	4	28
Combined (both EOG and VOG)	1	12
Source of normative values used*		
From own laboratory	80	N/A
From manufacturer of device	4	N/A
From previous publications	3	N/A
No normative values considered	15	N/A
Unclear	15	N/A

*EOG* electro-oculography, *VOG* video-oculography

*Numbers of healthy control subjects included were inconsistently reported only; thus, a total number of subjects is not available (N/A)

The specific quantitative parameters that were extracted, within each paradigm, varied across studies. For PEM, most studies focused on PEM gain (*n*=22 studies), whereas for SEM three different aspects were measured with similar frequency (saccadic latency [*n*=44 studies], peak eye velocity [*n*=47 studies] and amplitude/gain/accuracy [*n*=48 studies]). For saccadic intrusions (SIs), studies generally reported their frequency of occurrence without further specification. For spontaneous nystagmus (SN) and GEN, studies usually made a dichotomized distinction between SN/GEN being either present or absent. For qHIT analysis, all studies focused on qHIT gain.

### Normative Values in Healthy Controls

Where available, we extracted information on normative values (for the same experimental setting and setup) of healthy control populations (80 studies; *n*=1504 controls). For PEM, the average gains were similar for horizontal and vertical movements (see Figure S1 in Appendix 4), and both horizontal (*n*=13 studies) and vertical gain (*n*=4 studies) decreased with increasing peak stimulus velocity (*p* = 0.004, linear model with peak stimulus velocity and movement direction as fixed effect factors). For visually guided horizontal SEM, saccadic peak velocity increased with saccade amplitude (*p* < 0.001 across *n*=28 studies, see Figure S2 in Appendix 4), as expected. For saccadic amplitudes of 10° or lower, there was a large difference in the average normative value of saccadic peak velocity, gain, and latency (up to 40–70%; see Figures S2-5 in Appendix 4). For SIs, normative values were provided in only 4/24 studies that measured SI. These studies reported normative values on square-wave jerk (SWJ) frequency per minute and SWJ amplitude [22–25]. SWJ frequency varied substantially, ranging from 3.6±7.8 to 25.5±0.7 per minute. Normative values for nystagmus were only provided in 1/17 studies reporting on SN [26], and 2/22 studies reporting on horizontal GEN [25, 26], while no normative values were available for vertical SN (downbeat nystagmus [DBN], upbeat nystagmus). From the 17 studies that reported on qHIT, most used normative values proposed by the manufacturer of their device (see Table 4 for details), whereas only four studies developed their own normative values [21, 26–28].

### Oculomotor Parameters in Treatment Response Trials and Observational Studies

Both observational studies identified in our review reported on disease progression in SCA2 patients over a period of 12 months [19] and 5 years [20], respectively, and focused on horizontal visually guided saccades. Over a period of 5 years, SCA2 patients demonstrated a significant decrease in saccade peak velocity and saccade accuracy, whereas saccade latency increased. Faster progression rates of saccadic slowing were associated with larger CAG repeat length [20]. Importantly, the effect size of the within-subject longitudinal change for saccade peak velocity and latency was larger than Scale for the Assessment and Rating of Ataxia (SARA), the main clinical score, indicating higher statistical power to monitor the efficacy of a potential disease modifying therapy. In another study with a shorter follow-up period of 1 year, no significant decreases in saccade peak velocity were observed (with an average decrease over 1 year of 8°/s, *n* = 30 patients) [19].

Eleven studies used quantitative oculomotor assessments for evaluation of response to treatment [29–39]. Patient populations were diverse (ranging from acute autoimmune cerebellar disease, unspecified cerebellar ataxia, and autosomal-dominant cerebellar ataxia (ADCA) to episodic ataxia (EA) type 4, SCA2, FRDA, Niemann-Pick disease type C (NPC), and A-T) and sample sizes were often small (*n*=1-38). Oculomotor parameters used to assess treatment response included SI (*n*=2), visually guided saccades (*n*=5), PEM (*n*=2), rotational vestibulo-ocular reflex (rVOR) (*n*=3), SN (*n*=3), GEN (*n*=3), VOR decay time constant (Tc) (*n*=1), and optokinetic nystagmus (*n*=1). Significant treatment responses as assessed by oculomotor measurements could be detected in seven studies. This included reduction of SI in response to intravenous immunoglobulin in anti-GAD-antibody-positive cerebellar ataxia [30] and in response to memantine in ADCA [35]. Increased PEM gain and more stable eccentric gaze holding (i.e., reduced centripetal eye drift) were reported in EA4 under gabapentin treatment [31]. Furthermore, DBN in patients with various cerebellar pathologies [32] and rVOR decay Tc and gaze holding instabilities (SN, periodic alternating nystagmus [PAN]) in A-T [36] were reduced by 4-aminopyridine (4-AP). PAN was abolished and the rVOR decay Tc was normalized in a patient with anti-GAD-antibody positive cerebellar ataxia by baclofen [37] and saccadic latency was reduced by oral zinc sulfate supplementation in SCA2 [38].

### Recommendations for Quantitative Oculomotor Assessments in Hereditary Ataxia: Core Set of Paradigms and Parameters

To guide the design and implementation of quantitative oculomotor assessments in clinical studies of hereditary ataxia, we developed a core set of paradigms and parameters for validation in future studies. Consensus was achieved for the following core set of paradigms: (i) PEMs; (ii) SEMs; (iii) fixation stability, looking for SIs and SN; (iv) eccentric gaze-holding deficits, looking for GEN; and (v) rVOR using the qHIT. All these paradigms can be recorded with the participant seated on a chair (or in a wheelchair) using a single commercially available video-oculography (or possibly infrared oculography) device and a computer screen or headset (virtual reality or other built-in screen). Detailed information on the proposed paradigms, recommended recording conditions and parameters, are shown in Table 5.

**Table 5:** Core set of paradigms proposed and implementation details

Paradigm	Stimulus motion profile	Stimulus motion plane	Stimulus motion frequency/velocity	Stimulus motion amplitude (center to peak)	Stimulus appearance	Inter-stimulus interval	EM recording plane	Recording device/recording frequency	Number of eyes recorded	Recording duration/number of trials/cycles	Derived quantitative parameter	Comments
PEM	Sinusoidally moving	Minimal requirement: •Hor Recommended: •Hor and vert	0.1–0.4 Hz	10–20°	Target constantly on, in darkness or in dimly lit room*	NA	Minimal requirement: •Hor Recommended: •Hor and vert	VOG/IROG, alternatively: EOG^#^ ≧ 100Hz	Monocular (minimal requirement), binocular recommended	≥ 6 cycles per trial condition	Gain, velocity, saccadic intrusion	Sinusoidal movement preferred over constant vel stimulus
SEM	Visually guided saccades	Minimal requirement: •Hor Recommended: •Hor and vert	NA	Steps of 5–30°, random sequence for amplitude and direction (left/right, centripetal/centrifugal)	Target constantly on, in darkness or in dimly lit room*	Random duration (2–3s)^§^	Minimal requirement: •Hor Recommended: •Hor and vert	VOG/IROG, alternatively: EOG^#^ ≧ 100Hz	Monocular (minimal requirement), binocular recommended	≥ 10 trials per condition	Main sequence of saccades, saccadic peak velocity/latency/variability/gain	Memory-guided saccades and antisaccades: optional
SI	NA	NA	NA	NA	Target straight-ahead (in darkness or in dimly lit room*): •Target constantly on •Target off (with refixation flash of 50 ms every 2s)	NA	Minimal requirement: •Hor Recommended: •Hor and vert	VOG/IROG, alternatively: EOG^#^ ≧ 100Hz	Monocular (minimal requirement), binocular recommended	≥ 60s	Presence/absence of an inter-saccadic interval (ISI) and identification of type of SI based on extracted amplitude (deg)/duration (ms)/frequency (events/min) of SI	Recording together with SN paradigm. No ISI Ocular flutter (if restricted to hor plane) Opsoclonus (SI into multiple directions) With ISI Square-wave jerks Macrosaccadic oscillations (if large (5–15deg) amplitude))
SN	NA	NA	NA	NA	Target straight-ahead (in darkness or in dimly lit room*): •Target constantly on •Target off (with refixation flash of 50 ms every 2s)	NA	Minimal requirement: •Hor Recommended: •Hor and vert	VOG/IROG, alternatively: EOG^#^ ≧ 100Hz	Monocular (minimal requirement), binocular recommended	≥ 60s	SN slow-phase velocity (°/s), amplitude (°), frequency (Hz) and plane.	Recording together with SI paradigm
GEN/RBN	Sequence of target steps: baseline straight-ahead, eccentric position (GEN), straight-ahead (RBN)	Minimal requirement: •Hor Recommended: •Hor and vert	≦0.1Hz (i.e., ≧ 10s per position)	Target eccentricity: 5 to 30–40° (horizontal) and 5–20° (vertical optional)	Flashing target (50-ms flash every 2s) in darkness or in dimly lit room*	NA	Minimal requirement: •Hor Recommended: •Hor and vert	VOG/IROG, alternatively: EOG^#^ ≧ 100Hz	Monocular (minimal requirement), binocular recommended	NA	GEN/RBN slow-phase velocity (°/s), GEN/RBN decay over time, plane of GEN/RBN	
qHIT	Brief head impulses in the plane of the canal evaluated	Minimal requirement: •Hor plane Optional: •Vert plane (RAPL and LARP)	Peak head vel >150°/s (hor plane) >120°/s (vert plane)	10–20° (starting from straight-ahead position or from eccentric head position)	Constant target (light or darkness)	≧5s	Minimal requirement: •Hor Recommended: •Hor and vert	VOG ≧ 100Hz	Monocular (minimal requirement), binocular recommended	10-20 valid head impulses per canal	HIT gain, optional: cumulative saccadic amplitude	Calculation of gain: instantaneous (regression) gain at a given time point (e.g., 60ms) or area-under-the-curve gain

*Avoiding direct exposure to sunlight and with luminance measured and kept within a narrow range

^§^Higher upper limit may be needed for selected patient populations

^#^If VOG/IR not available and recordings restricted to horizontal eye movements

*Abbreviations*: *EOG* electro-oculography, *GEN* gaze-evoked nystagmus, *HIT* head-impulse test, *hor* horizontal, *IROG* infrared oculography, *ISI* intersaccadic interval, *LARP* left-anterior-right posterior, *NA* not available, *NS* not specified, *PEM* pursuit eye movements, *qHIT* quantitative head-impulse test, *RAPL* right-anterior-left posterior, *RBN* rebound nystagmus, *SEM* saccadic eye movements, *SI* saccadic intrusions, *SN* spontaneous nystagmus, *vert* vertical, *VOG* video-oculography

### Guidelines for Recording Setup, Calibration, and Minimal Requirements

Standardization of data acquisition with clearly defined minimal requirements is key to achieve reliable high-quality recordings, especially for multicenter studies. Thus, the consensus process included detailed technical recommendations on various aspects, including preferred hardware used for eye movement recordings, necessary calibration procedures, and prior clinical assessments to determine whether binocular recordings are possible.

### Ocular Alignment

Before initiating a recording session, we recommend that both an assessment for ocular alignment and a calibration should be performed. To assess for ocular alignment, both a cover/uncover test and an alternate cover test should be done (and recorded if binocular recording devices are used) while looking straight-ahead, measuring any ocular deviation angle [40, 41]. The alternate cover test allows the identification of any skew deviation (i.e., vertical divergence or vertical tropias), and the cover/uncover test can reveal horizontal tropias. Note that horizontal phorias seen during the alternate cover test (i.e., always only one eye viewing) are frequently seen and require no further adjustments. In patients with horizontal or vertical tropias, monocular recordings should be performed with the non-recorded eye covered.

### Recording Setup

The use of commercially available video-oculography devices is strongly recommended with a minimal recording frequency of 100Hz, which is critical for fast eye movements [42] as saccades (taking into account published recommendations for the recording of saccades [43]) and the vestibulo-ocular reflex. While monocular recordings are considered acceptable as a minimal requirement, we recommend binocular recordings to allow coverage of a larger field of view and for controlling ocular alignment. If performing monocular recordings (e.g., due to VOG restrictions or ocular misalignment), the eye not recorded should be covered to make sure the recorded eye is the viewing eye. Likewise, recordings of both horizontal and vertical eye movements are recommended to allow a better characterization of ocular abnormalities such as vertical saccade slowing or vertical (down-beating) spontaneous nystagmus.

### Calibration

Calibration is essential to ensure good data accuracy and precision. During calibration, the geometric characteristics of the subject’s eye are estimated and incorporated into the calculation of the subject’s gaze point (see, e.g., [44]). Subjects are typically asked to look at specific points (or calibration dots) in the screen that rapidly move from one position to another. For VOG systems, the calibration sequence is generally optimized for each device and provided by the manufacturer. It is important to note (and anticipate) that some ataxia patients may not be able to execute calibration procedures that have been designed for healthy volunteers. In fact, slow eye movements, fixation instabilities, or limited range of movement in some patients may require adapting the default calibration procedures. For example, some default calibration procedures jump quickly from one calibration point to the next, and participants with severely reduced saccade velocities (e.g., due to severely abolished saccade generation in NPC) or patients who require corrective saccades to reach the calibration points may not be able to perform a correct calibration with the default timing. Such patients may require a calibration sequence based on pursuit rather than saccades. There is no optimal one-fits-all calibration procedure given the variable pattern of oculomotor deficits across hereditary ataxias. Thus, we recommend that characteristics of the population of interest are reviewed before initiating a study. Calibration may potentially also require acquisition of some pilot data prior to the study. Additionally, we recommend that the calibration performance parameters such as accuracy and precision are recorded. Such quality control measures can be important to interpret discrepancies among study subjects, for example, when comparing datasets from different sites that differ in data quality [8, 45].

### Standardized Stimulation Procedures

A standardized stimulation procedure is recommended for all eye movement recordings. For qHIT recordings, stimulation sequences and quality control criteria are typically available from the manufacturer of the device. Other eye movement types (PEM, SEM, SN/GEN, SI) may require designing customized stimulation sequences. When using different devices across sites for multicenter studies, efforts should be made to standardize the acquisition procedure, and use—when possible—the same stimulus sequence and acquisition protocol. Practice trials should be considered and implemented whenever possible before starting the actual test sequence. Detailed guidelines on recommended stimulus parameters such as stimulus motion, the number of trials, and the recording duration can be found in Table 5.

## Discussion

Based on a systematic and comprehensive review of the literature, we recommend a prioritized set of oculomotor paradigms and derived quantitative parameters for further validation in multicenter clinical trials in hereditary ataxia: (i) pursuit eye movements, (ii) saccadic eye movements, (iii) fixation (including spontaneous nystagmus and saccadic intrusions), (iv) eccentric gaze holding (gaze-evoked nystagmus), and (v) the rotational vestibulo-ocular reflex as assessed by the head-impulse test. We complement this recommendation with a specific technical guideline for data acquisition to facilitate the standardization of measurements. All proposed parameters have demonstrated discriminatory power, correlation with disease severity, and/or intra-individual sensitivity to change in previous studies (as discussed in detail in the companion paper [46]), and all proposed paradigms are feasible for multicenter trial deployment since they may be measured with simple, commercially available, relatively affordable and portable recording systems. Together, the paradigms and parameters comprehensively can capture for potential pathology in the entire brain circuitry and vestibular system which underlies eye movement abnormalities in hereditary ataxia. This includes the cerebellum, brainstem nuclei, and the cerebral cortex. We consider our recommendations as a core set of paradigms and parameters, which may require adaptation to the study population under examination (e.g., specific ataxia genotypes), and that can be adjusted (adding, e.g., stimulus movement along the vertical plane or different types of saccades) as required. In the framework of the Ataxia Global Initiative, we particularly consider our recommendations an evidence-based prioritization of paradigms and parameters for longitudinal validation studies needed to show their value as digital-motor outcome measures in clinical trials.

### Selection of Paradigms and Devices for Recording Eye Movements in Hereditary Ataxia

Recording device selection is critical for the success of eye movement recording, as for example a 30-Hz recording rate will not capture fast eye movements as saccades appropriately. To improve handling, recording quality, and comparability in multi-site trial settings, we recommend the use of commercially available, certified devices that have an established track record in the field. Devices with high-resolution, high recording frequency, and low noise levels are required to achieve sufficient eye movement recording quality [8]. Recording of both horizontal and vertical eye movements is preferred. Low-quality eye trackers and smartphones are currently not recommended, since they lack the accuracy, precision, and sampling frequency required to robustly measure oculomotor parameters. With further technological advances in smartphone cameras, however, they may prove useful in the near future. The use of electro-oculography devices may be sufficient for selected paradigms (including horizontal SEM or horizontal PEM), but they have higher noise levels, baseline drift, and lid artifacts, especially for vertical eye movements [47]. At the same time, magnetic scleral search coil recordings—still considered the gold standard with regard to quality and versatility of eye movement recordings—are not feasible for multicentric clinical trials due to their technical and financial demands, and are also associated with increased participant burden [48].

Full access to the raw data and support of individualized stimulus protocols is critical. This can be essential to identify and correct potential data quality issues, or to perform additional sensitivity analyses. For example, being able to review the raw eye movement traces can help identify and remove artifacts, which may otherwise contribute to noise in the data. For multicenter trials, the optimal proposal would be to use identical devices and setups at all sites. However, this is often not feasible, in which case employing different devices across sites could be acceptable, but it is important to seek consistent acquisition conditions, paradigm and operating procedures. Here, it is important to take into account the technical characteristics of the included devices, and the potential biases associated with each specification. For instance, sampling frequency impacts the estimation of saccade duration or velocity [42] and thus an important parameter to keep in mind when choosing the eye movement recording device to be used.

### The Importance of Normative Values

The impact of normative values depends on the study design. While for longitudinal observational studies or for treatment trials intra-individual comparisons with either baseline measurements at the study initiation or before treatment are made, normative values will play a more prominent role in case-control studies. Differences in normative values identified in the studies we included may have distinct underlying causes including data smoothening (especially for saccades), synchronization errors, distraction of subjects, fatigue, stimulus parameter variations including stimulus size, stimulus brightness, background light, recording type (VOG, EOG), recording device, and a speed-accuracy tradeoff. Overall, the range of normative values was considerable—and often they were not reported. Across the literature, there are numerous reports of normative values for horizontal PEM gains and horizontal visually guided saccade latency, peak velocity, and gains. In contrast, there are few studies reporting healthy control values for vertical saccades, memory-guided saccades, anti-saccades, SI, SN, and GEN.

When using video-oculography, however, using normative values provided by the manufacturer of the device seems justified for the qHIT. Noteworthy, normative values may vary among measurement systems used and comparative studies have been published. For the qHIT, normative values depend on the peak head velocity values; thus, the application of head impulses needs training and experience, as well as good-quality control criteria to maintain individual impulses within the intended velocity range. Whereas for horizontal semicircular canals normative values are very similar for different devices (e.g., EyeSeeCam [Interacoustics, A/S, Denmark], ICS Impulse goggles [Natus, USA]), stronger discrepancies have been reported for the vertical canals [49, 50].

### Study limitations, areas of limited knowledge, future directions

The oculomotor parameters proposed here were derived from paradigms used in previously published studies, and were the result of extensive discussion and consensus among the members inside and outside of this working group. The full potential of these parameters across ataxia genotypes, however, has not yet been revealed and other oculomotor parameters not considered here may be valuable as well. Prospective, disease-specific longitudinal validation studies measuring the within-subject progression and heterogeneity of this set of parameters are now needed to fully assess their potential as digital-motor biomarkers in clinical trials in hereditary ataxia. The heterogeneity in the patient populations of the included studies (ranging from genetically proven ataxias to sporadic or acquired ataxias) and the limited data for various oculomotor paradigms and derived parameters are important limitations of this systematic review. With the majority of studies in our systematic review being of low overall study quality with regard to reporting oculomotor function in hereditary ataxia, there is a need for more high-quality studies in this field.

From a regulatory perspective, clinical outcome measures not only require adequate metric properties (i.e., sensitivity to change), but must also be functionally meaningful to the patient [51]. However, there are currently no established clinical or performance measures capturing functional impairment by oculomotor dysfunction in hereditary ataxia. While the dynamic visual acuity provides a functional assessment of the integrity of the rotational vestibulo-ocular reflex, this paradigm has been considered in a single study only in our literature review [52]. Other potentially suitable measures include assessments of visual stability, i.e., the amount of visual impairment by fixation instability as, e.g., SN, GEN, and SI or reading performance [53]. Future validation studies of quantitative oculomotor parameters in hereditary ataxia must include such clinician-reported or patient-reported outcome measures of oculomotor function to effectively aim for trial readiness.

## Conclusions

Based on a systematic literature review, we have selected a core set of quantitative oculomotor parameters for capturing eye movement abnormalities in (hereditary) ataxias. These parameters now require further, disease-specific prospective validation in both observational and clinical studies focusing on their reliability, validity, sensitivity to change, and eventually functional meaningfulness to patients. We have provided detailed measurement and analysis guidelines based on previously published studies in the field and approved these recommendations through a multi-step review process within our working group. The protocol should be tailored to each specific study and population, pruning or adding to the core paradigms that we have proposed here. This will aid the implementation and interpretation of oculomotor parameters in clinical and observational trials and thus advance our understanding of the evolution of oculomotor and vestibular network dysfunction in hereditary ataxias. Importantly, the use of commercially available, mobile recording devices with recording frequencies above 100Hz and based on video-oculography is strongly recommended.

### Supplementary information

ESM 1

## Data Availability

The data that support the findings of this study are available from the corresponding author upon reasonable request.
